# Loss of chromosome Y (LOY) in blood cells is associated with increased risk for disease and mortality in aging men

**DOI:** 10.1007/s00439-017-1799-2

**Published:** 2017-04-19

**Authors:** Lars A. Forsberg

**Affiliations:** Science for Life Laboratory, Beijer Laboratory of Genome Research, Department of Immunology, Genetics and Pathology, Uppsala University, Uppsala, Sweden

## Abstract

Recent discoveries have shown that harboring cells without the Y chromosome in the peripheral blood is associated with increased risk for all-cause mortality and disease such as different forms of cancer, Alzheimer’s disease, as well as other conditions in aging men. In the entire world, the life expectancy of men is shorter compared to women, a sex difference that has been known for centuries, but the underlying mechanism(s) are not well understood. As a male-specific genetic risk factor, an increased risk for pathology and mortality associated with mosaic loss of chromosome Y (LOY) in blood cells could help to explain that men on average live shorter lives compared to women. This review primarily focuses on observed associations between LOY in blood and various diseases in aging men. Other topics covered are known risk factors for LOY, methods to detect LOY, and a discussion regarding mechanisms such as immunosurveillance, that could possibly explain how an acquired mutation in blood cells can be associated with disease processes in other organs.

## Introduction

**Fig. 1:**
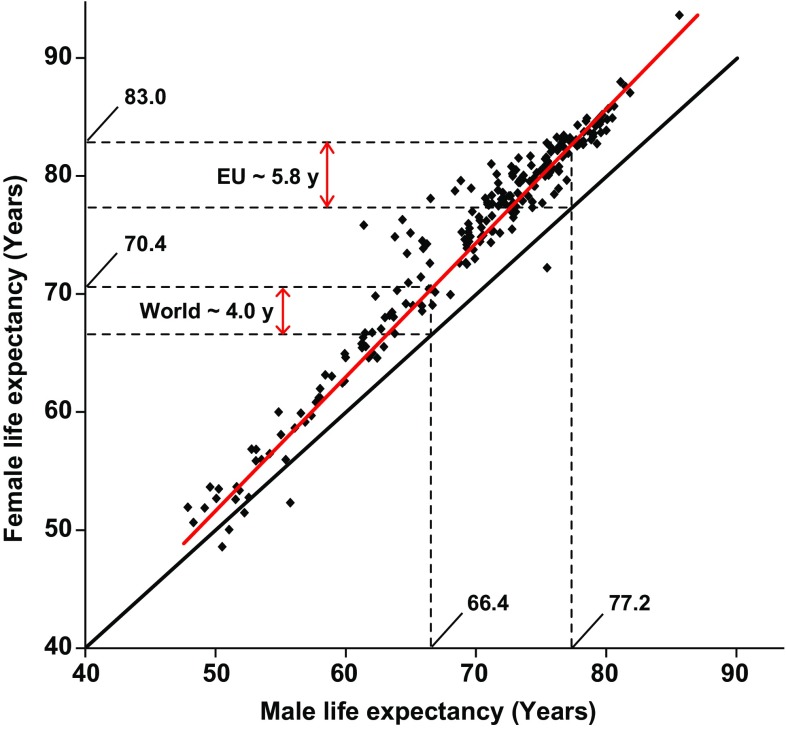
Men live on average shorter lives compared to women. Each *dot* shows data from one nation or human population. The *red line* represents the observed difference in lifespan and the *black line* represents a null hypothesis with no difference in longevity between the sexes. The *dotted lines* mark the male and female life expectancies globally and in the European Union (EU). Data from *The World Factbook 2013* on male and female life expectancy at birth in different human populations in 2013 (https://www.cia.gov/library/publications/the-world-factbook/)

It has been known for centuries that men on average live shorter than women (Nathanson 1984 and references therein). This sex bias is present in the entire world and men live on average about 4 years shorter compared to women (66.4 and 70.4 years for men and women, respectively) and the difference is larger in populations with longer life expectancy (Fig. 1). For example, the sex difference in the EU is about 5.8 years (77.2 and 83.0 years for men and women, respectively). This pattern, i.e., a larger difference in populations with longer life expectancy, suggests that the underlying factor(s) is stronger in populations with a larger proportion of the total mortality related to age-associated diseases. Recent discoveries discussed here, regarding pathological effects from a male-specific genetic risk factor, i.e., loss of chromosome Y (LOY) in blood cells, may help to explain the observed sex difference in longevity.

In the present review, LOY is primarily discussed as a mosaic event, with a mixture of cells with and without the Y chromosome in the peripheral blood of normally aging men. At the cellular level, LOY is a binary state, either a cell has a Y chromosome or not. Microdeletions affecting chromosome Y and associated phenotypic consequences, such as spermatogenic failure in men (Kuroda-Kawaguchi et al. 2001), are a different event. On the level of an individual, LOY in blood cells is a continuous estimate ranging from 0 to 100% of blood cells affected, with all or none of the blood cells carrying a Y chromosome, respectively. Current methods for detection of LOY in a sample are primarily based on quantifying a lower than expected abundance of DNA derived from the chromosome Y, in relation to reference loci from other parts of the genome. Commonly used platforms include SNP arrays, different forms of quantitative PCR, as well as next-generation sequencing (NGS). BOX 1 describes an approach used to detect LOY from the intensity data generated in SNP-array experiments. For scoring individual men with or without LOY, first requires the definition of a threshold of mosaicism to be used as a cut-off level. Hence, the threshold should to be low enough to pick up low-level mosaicism, but high enough to avoid false-positive calls, as further explained in BOX 1.

For example, using a threshold of ≥10% of affected cells in three independent cohorts studied by SNP arrays, a recent study demonstrated that about 7, 14, 18, and 20% of men were affected in the ages of ≤65, 66–75, 76–85, and ≥86, respectively (Dumanski et al. 2016). These data show that LOY accumulates with age in the studied cohorts and that LOY is the most common acquired human mutation during life in normal peripheral blood of men, and it is affecting ~1.6% of the human genome (Forsberg et al. 2017).

In addition to age, smoking was described as an important risk factor for being affected with LOY in blood cells (Dumanski et al. 2015). Analyses adjusting for age and other confounders including 6014 men from three independent cohorts showed that current smokers had up to four-fold increased risk of LOY in blood cells. A dose–response effect was observed in current smokers, since smokers with LOY had been smoking significantly more pack-years compared to smokers without LOY. Furthermore, the effect from smoking on LOY in blood was transient, as previously regular smokers on average displayed a similar level of LOY as the never smokers. This result could imply that clones with LOY disappear from the blood subsequent to smoking cessation. An independent analysis including 13,789 men from three studies recently replicated the association between LOY and smoking (odds ratio, OR = 2.35) (Zhou et al. 2016). This paper also found a transient effect, since the strength of the association between smoking and risk for LOY weakened with years after cessation.

## LOY in blood cells and risk for disease

A frequent loss of chromosome Y in hematopoietic cells of aging men was first described more than 50 years ago (Jacobs et al. 1963; Pierre and Hoagland 1972), but the pathological consequences of LOY have been elusive. For many years, the consensus was that LOY was phenotypically neutral and related to normal aging processes (UKCCG 1992; Stone and Sandberg 1995; Wiktor et al. 2000; Wong et al. 2008). However, some recent analyses suggest the opposite that LOY in blood cells could be directly involved with disease processes taking place in various organs. It is important to separate the effects from LOY in blood cells of normally aging men, primarily discussed in this paper, from the nullisomy Y observed frequently in other tissues and tumors in various organs. For example, the Y chromosome is frequently lost in cancer genomes with frequencies ranging from 15 to 80% of cases in different types cancers (Hunter et al. 1993; Park et al. 2006; Bianchi 2009; Duijf et al. 2013). The separation between pathogenic effects from LOY in non-transformed blood cells from the effects of nullisomy Y in oncogenic clones is important, in particular in studies of hematological malignancies. For this reason, the following sections discuss associations between LOY in blood cells and risk for diseases grouped into hematological disorders, non-hematological tumors, Alzheimer’s disease, as well as other diseases.

### LOY in blood cells and risk for hematological disorders

There is no clear consensus today regarding the associations between LOY in blood and risk for various hematological malignancies. Some reports describe associations with LOY and worse prognosis in leukemias such as acute myelogenous leukemia (AML) (Holmes et al. 1985), cronic myeloid leukemia (CML) (Lippert et al. 2010), and chronic lymfocytic leukemia (CLL) (Chapiro et al. 2014). In other reports, the prognostic association of LOY for survival in hematologic disease such as myelodysplastic syndrome (MDS) appears to be neutral or even favorable (Wiktor et al. 2000). For example, MDS patients with LOY as the only cytogenetic aberration showed an unexpectedly long overall and AML-free survival (Greenberg et al. 1997). Another report suggested that LOY in bone marrow cells from elderly could not be used as predictor of hematological malignancy (UKCCG 1992). Remarkably, some studies indicate that the association is robust only in patients with LOY in a very high proportion of blood cells (Wiktor et al. 2000; Wong et al. 2008), and this indicates that a key for investigating LOY in hematological disorders would be to analyze LOY in relevant cells types in the blood. Such analysis was described in a recent paper comparing level of LOY in CD34+ blood cells (involved in MDS) with level of LOY in CD3+ T-cells (not involved in MDS) in MDS patients and controls of different ages (Ganster et al. 2015). The percentage of CD34+ cells with LOY was higher in MDS patients compared to elderly control men without hematologic diseases. Furthermore, LOY was not detected in CD3 + cells of young men, but it was found in CD3+ cells of elderly men without hematologic diseases, as well as in elderly MDS patients. Together, these data suggest that LOY in blood has an age-related basis but is also associated with MDS. A take-home message so far from the field of hematological malignancies is that LOY in blood could be described in terms of both an age-dependent event as well as a potential marker of neoplastic clones in blood.

### LOY in blood cells and risk for non-hematological tumors

Associations between LOY in blood cells and increased risk for hematological malignancies described above may not be surprising. However, more unexpected results describe associations between mosaic LOY in blood and increased risk for various non-hematological tumors. First, in a prospective study called ULSAM including naturally aging men, it was found that men with LOY in a substantial proportion of their peripheral blood cells had increased risk for all-cause mortality in adjusted Cox proportional hazard regression (hazard ratio, HR = 1.91) (Forsberg et al. 2014). Men with LOY in blood cells lived on average 5.5 years after blood sampling, compared to about 11 years average survival in men without LOY in blood. This association was replicated in the independent PIVUS cohort (HR = 5.24). Further analyses in ULSAM showed that men with LOY in blood had an increased risk for cancer diagnosis (HR = 2.47) as well as increased risk from mortality in various cancers outside the hematopoietic system (HR = 3.76). Men with LOY in blood were affected with a similar spectrum of cancers compared to men without detectable LOY in blood cells. The blood samples analyzed were collected from participants between the ages of 70 and 83 years and the follow-up time after the blood sampling was up to 20 years. No men with cancer before sampling or hematological malignancy were included in any of the analyses. Individual LOY status was scored from the log *R* ratio (LRR) generated by Illumina SNP-array genotyping, as explained in BOX 1. Subsequent whole-genome NGS experiments validated the LOY calls with 100% concordance.

In a more recent study, association between LOY in peripheral blood and risk for cancer diagnosis outside the hematopoietic system was validated in 101 colon cancer patients, 70 prostate cancer patients, and 93 healthy controls, by means of LOY scoring with quantitative fluorescent PCR (Noveski et al. 2016). The relative amount of Y chromosome derived DNA in blood cells was significantly lower in cancer patients compared to controls, when analyzed both together and separately. Multivariate logistic regression analysis indicated LOY to be a better predictor of cancer outcome compared to age. In summary, this relatively small case–control study describes a link between cancer presence and mosaic LOY in peripheral blood of men affected by the two most common solid tumors in men.

Another recent study investigated whether LOY in blood and buccal samples was associated with non-hematological cancer in three cohorts including 8679 cancer cases and 5110 controls (Zhou et al. 2016). Comparing the levels of LOY in cases and controls showed significantly higher levels of LOY in prostate cancer patients (adjusted OR = 1.35) as well as in bladder cancer patients (adjusted OR = 1.47) but no significant difference in lung cancer patients and controls. Furthermore, this study also reported some less conclusive results regarding associations between LOY and cancer risk and survival. It is not clear if the use of DNA from two different tissues, i.e., ~70% of samples were blood samples and ~30% came from buccal samples, could explain the weaker associations observed in this study, as further discussed in the section on immunosurveillance below.

### LOY in blood cells and risk for Alzheimer’s disease

A link between LOY in peripheral blood cells and risk for Alzheimer’s disease (AD) has also been reported (Dumanski et al. 2016). Analyses included 754 men diagnosed with AD and 2464 controls from three independent cohorts. In a case–control study, logistic regression showed after fitting the effects from *APOE*-genotype and age that males with AD diagnosis had higher degrees of LOY mosaicism compared to controls (adjusted OR = 2.80). Furthermore, in two prospective studies, men with LOY at blood sampling had a greater risk to be diagnosed with AD during the follow-up time (HR = 6.80). It is interesting that LOY in blood is associated with increased risks for both cancer and AD, and it is possible that these outcomes act as competing risks. A stronger association between LOY in blood and increased risk for the two conditions was found after excluding men with the other outcome from control groups, supporting the suspicion of competing risks (Dumanski et al. 2016).

### LOY in blood cells and other diseases

Loss of the Y chromosome in blood cells has also been suggested to be involved in the pathogenesis of rare autoimmune conditions, such as autoimmune thyroiditis (Persani et al. 2012) and primary biliary cirrhosis (Lleo et al. 2013). For example, in an analysis of LOY in blood cells, using FISH in men diagnosed with autoimmune thyroiditis, it was found that 31 male patients had on average a higher level of mosaicism compared with 88 healthy controls (Persani et al. 2012). The level of LOY in blood was associated with age in both groups and the level of LOY mosaicism increased at a higher rate in the patient population compared to the healthy controls.

## LOY in blood cells associated with disease processes in other organs

The studies discussed above describe associations between LOY in blood cells and increased risk for various diseases (Holmes et al. 1985; Lippert et al. 2010; Persani et al. 2012; Lleo et al. 2013; Chapiro et al. 2014; Forsberg et al. 2014; Ganster et al. 2015; Dumanski et al. 2016; Noveski et al. 2016; Zhou et al. 2016). A critical question to address is how could LOY affecting non-cancerous cells in the peripheral blood can be associated with disease processes in other organs, such as neoplastic proliferation of cells leading to tumors in other organs or neurodegenerative processes in the central nervous system? So far, the causal mechanism(s) behind associations of LOY in blood and disease in other tissues are not well known, and different possibilities are considered. One hypothesis is that general LOY mosaicism in human tissues could act as a marker of biological aging and thereby be linked with age-associated diseases. However, results from our lab show that not all age-related diseases are associated with LOY in blood. For example, we have tested, but found no associations between LOY in blood and diseases such as chronic obstructive pulmonary disease (COPD) and Parkinson’s disease (unpublished). If age per se directly explained the association between LOY in blood and increased risk for age-associated diseases, we would expect associations between LOY in blood and all diseases that are more common in elderly.

Usually, age is included as a confounder in the analysis of LOY and its associations with various outcomes. Another approach to further discriminate between the increased risks for disease conferred by age from risk mediated by LOY would be to neutralize the age-mediated risk by studying the effect from LOY in groups of men with similar ages. We used this approach in a publication that describes an association between LOY in blood and AD (Dumanski et al. 2016). First, a Cox proportional hazard regression adjusting for confounders on a merged data set (i.e., men in the ULSAM and PIVUS cohorts between 70 and 84 years of age, *n* = 1607) found that age as well as level of LOY were significantly associated with increased risk for AD (HR = 1.24 and 6.80, respectively). We then performed two additional analyses using Cox regression, adjusting for the same confounders as before. The first model included only men between the ages 70–75 years of age and the second model was performed with men between 75 and 80 years of age. In these two analyses, the previously significant age effect, found in the model including men of all ages, could not be observed. In contrast, the association between LOY and an increased risk for AD was robust also in these analyses, including subsets of men at ages 70–75 (HR = 25.82, *n* = 1047) as well as between ages 75–80 (HR = 9.92, *n* = 278). Hence, these results suggest that LOY in blood cells is a risk factor for AD that is independent from the increased risk mediated by age.

## Reduced ability to fight disease because of LOY in immune cells

We have previously discussed the hypothesis that disrupted immune system functions might be involved in the causality behind the associations between LOY in blood cells and increased risk for diseases in other organs (Forsberg et al. 2014, 2017 Dumanski et al. 2015, 2016). A normally functioning immune system includes actions such as immunosurveillance that combat defiant cells in various tissues and of different types in the body. If LOY in blood cells leads to defective immunosurveillance performed by immune cells in blood, it might pose a possible link between observed associations and underlying causality. The hypothesis is that normal immune cell functions, such as the suppression of tumor cells in other organs (Dunn et al. 2002), are negatively affected in cells without the Y chromosome. Interestingly, a deficient immunosurveillance in the CNS, that normally eliminates abnormal cells related to an AD phenotype in the brain, has previously also been proposed as a mechanism in AD development (Simard et al. 2006; Schwartz and Shechter 2010; Ousman and Kubes 2012). The immunosurveillance hypothesis is attractive, since it could neatly explain the mechanism(s) behind associations between LOY in blood cells and increased neoplastic proliferation of cells into tumors in the entire body, as well as increased neurodegenerative processes in the central nervous system leading to AD development.

Immunosurveillance is a process performed by the immune cells, and analyses of associations between LOY and disease, and possible underlying causality by reduced immune functions, require studies of cells representing the immune system, such as peripheral blood cells. In a recent paper discussed above (Zhou et al. 2016), analyses were based on LOY in two different organs, i.e., ~70% were blood samples and ~30% were buccal samples. If a disturbed immunosurveillance in blood cells with LOY constitutes a link to an increased risk for disease in other organs, then mixing data from blood and buccal cells would tend to dilute an association, since buccal cells would not be part of the immune system. Hence, to study further the hypothesis that LOY might be involved in disturbing normal immunosurveillance processes, a way forward will be to focus analysis on LOY scored from blood samples.

## Conclusions

The most common acquired human mutation during life is the loss of the Y chromosome (LOY) in blood cells of men and it is associated with age and smoking. Affected men were found to have an increased risk for mortality as well as an increased risk for various disorders, possibly via defective immune functions of the affected blood cells. Older men with LOY in blood were reported to live on average only half as long compared to men without LOY in blood. As a male-specific genetic risk factor, LOY might help explain that men in the entire world live on average shorter lives compared to women. It is possible that LOY testing of middle-aged and aging men could help to reduce this sex difference, by earlier discovery of early phases of disease and more effective preventive medical practice.

## BOX 1 detection and scoring of LOY from SNP-array data

SNP-array genotyping is commonly used in genome-wide association studies (GWAS), and several manufacturers provide arrays with a varying number of probes located at the Y chromosome that can be used for LOY detection. For example, the degree of LOY in a subject can be estimated from Illumina SNP-array data, by calculating the median of the log *R* ratio (LRR) values of the probes located in the male-specific region of chromosome Y (MSY, chrY: 2,694,521–59,034,049, hg19/GRCh37) (Forsberg et al. 2014). This calculation generates a continuous estimate of the level of LOY in a sample (i.e., the mLRR-Y). The LRR is a measure of DNA copy number, generated during the genotyping along with the B Allele Frequency (BAF) as well as the SNP calls. An mLRR-Y value close to zero indicates a normal state, whereas values that are more negative indicate increasing degree of blood cells without the Y chromosome. Statistical analyses can be performed directly using the continuous mLRR-Y, but to score individual men with or without LOY, require the definition of a suitable threshold. Hence, a lower threshold (i.e., more negative mLRR-Y values) represents LOY in a higher degree of cells. In principle, different mLRR-Y values could be used as threshold to score LOY, each reflecting specific levels of mosaicism. For example, we have shown that mLRR-Y values below −0.4 represent LOY in at least ~35% of cells and that this level is suitable for studying the pathological consequences of LOY (Forsberg et al. 2014; Dumanski et al. 2016). Furthermore, we have developed a method to define an mLRR-Y threshold for confident detection of lower level mosaicism. First, the total variation seen in the mLRR-Y distribution from a SNP-array data set of aging men typically consists of two signals, i.e., one from the LOY events (as a tail on the negative side of the total mLRR-Y distribution) as well as variation originating from experimental factors (distributed around a local regression peak close to zero). To define the extent of this non-LOY-related variation and to set a threshold, this experimentally induced variation can be estimated by reflecting the data in the positive tail of the total mLRR-Y distribution, into a simulated negative tail, assuming that the experimental variation in mLRR-Y is distributed in a non-skewed fashion. This operation generates a new distribution, representing the variation of the data that originate from non-LOY factors. The lower 99% confidence limit of this distribution can be used as a threshold for confident scoring of low-level mosaicism. Recent analyses have shown that this approach allows robust and reproducible detection of LOY when it occurs in ≥10% of the nucleated cells in a blood sample (Dumanski et al. 2015, 2016). Furthermore, it have been shown that bad-quality SNP-array data can introduce a bias in the estimation of LOY and it is, therefore, important to assess the quality of SNP-array experiments at the sample level (Dumanski et al. 2016). The genotyping facilities regularly control the SNP-call quality, but the quality of LRR and BAF data tracks should be considered as well. For example, one of the SNP-array manufacturers recommends using the LRR standard deviation as strict quality criteria. A high LRR standard deviation reflects poor genotyping quality and only experiments with a value below 0.28 should be considered as high quality (see https://www.illumina.com/content/dam/illumina-marketing/documents/products/appnotes/appnote_cnv_loh.pdf). To avoid false-positive as well as false-negative LOY calls, the data generated by discovery platforms should preferably be validated using alternative technology, at least in a subset of the material.
